# Hypoglycemia incidence and awareness among insulin-treated patients with diabetes: the HAT study in Brazil

**DOI:** 10.1186/s13098-018-0379-5

**Published:** 2018-11-21

**Authors:** Rodrigo Nunes Lamounier, Bruno Geloneze, Silmara Oliveira Leite, Renan Montenegro, Lenita Zajdenverg, Milene Fernandes, Fabiano de Oliveira Griciunas, Mariana Narbot Ermetice, António Roberto Chacra, HAT Brazil study group, Adriana Costa e Forti, Ana Priscila Soggia, Daniela Espíndola Antunes, Flavia Coimbra Pontes Maia, Giselle Fernandes Taboada, Hugo Roberto Kurtz Lisboa, Jorge de Faria Maraschin, Jorge Luiz Gross, Manuel dos Santos Faria, Márcio Antonio Pereira, Maria Elizabeth Rossi da Silva, Miguel Nasser Hissa, Mirian Takahashi, Rosane Kupfer, Tania Maria Bulcão Lousada Ferraz

**Affiliations:** 1 Centro de Diabetes de Belo Horizonte, Belo Horizonte, MG Brazil; 2 Laboratory of Investigation on Metabolismo and Diabetes (LIMED), University of Campinas-UNICAMP, Campinas, SP Brazil; 3 Hospital Cruz Vermelha, Curitiba/PR. Centro de Diabetes de Curitiba, Curitiba, PR Brazil; 4 Faculdade de Medicina da Universidade Federal do Ceará, Fortaleza, CE Brazil; 5 Universidade Federal do Rio de Janeiro, Rio de Janeiro, RJ Brazil; 6 Eurotrials, Scientific Consultants SA, Lisbon, Portugal; 7 Novo Nordisk A/S, São Paulo, SP Brazil; 8 Departamento de Medicina da Universidade Federal de São Paulo-UNIFESP, São Paulo, SP Brazil

**Keywords:** Diabetes mellitus type 1, Diabetes mellitus type 2, Hypoglycemia, Insulin therapy

## Abstract

**Background:**

Hypoglycemia affects patient safety and glycemic control during insulin treatment of both type 1 (T1DM) and type 2 diabetes mellitus (T2DM). The Hypoglycemia Assessment Tool study in Brazil aimed to determine the proportion of patients experiencing hypoglycemic events and to characterize patient awareness and fear about hypoglycemia, among insulin-treated T1DM or T2DM patients.

**Methods:**

This was a non-interventional, multicenter study, with a 6-month retrospective and a 4-week prospective evaluation of hypoglycemic events. Patients completed a questionnaire at baseline and at the end of the study, and also a patient diary. The answers ‘occasionally’ and ‘never’ to the question ‘Do you have symptoms when you have a low sugar level?’ denoted impaired hypoglycemia awareness. Fear was reported on a 10-point scale, from ‘not afraid at all’ to ‘absolutely terrified’.

**Results:**

From 679 included patients, 321 with T1DM and 293 T2DM, median age of 33.0 and 62.0 years, 59% and 56% were female, and median diabetes duration was 15.0 and 15.0 years, respectively. Median time of insulin use was 14.0 and 6.0 years. During the prospective period, 91.7% T1DM and 61.8% T2DM patients had at least one hypoglycemic event. In the same period, 54.0% T1DM and 27.4% T2DM patients had nocturnal hypoglycemia, 20.6% T1DM and 10.6% T2DM patients had asymptomatic hypoglycemia, and severe events occurred in 20.0% and 10.3%, respectively. At baseline, 21.4% T1DM and 34.3% T2DM had hypoglycemia unawareness. The mean score of hypoglycemia fear was 5.9 ± 3.1 in T1DM and 5.4 ± 3.9 in T2DM. The most common attitude after hypoglycemic events were to increase calorie intake (60.3%) and blood glucose monitoring (58.0%) and to reduce or skip insulin doses (30.8%).

**Conclusions:**

Referred episodes of hypoglycemia were high, in both T1DM and T2DM insulin users. Patient attitudes after hypoglycemia, such as reduction in insulin and increase in calorie intake, can affect diabetes management. These findings may support clinicians in tailoring diabetes education and insulin treatment for patients with diabetes, in order to improve their glycemic control while reducing the risk of hypoglycemic events.

## Background

Diabetes mellitus is a major cause of morbidity and mortality worldwide, as a result of its impact on cardiovascular system, eyes, kidneys, and nerves [1]. Optimizing blood glucose control is demanding, since it requires balancing the need for glycemic control with the risk of hypoglycemia [2–4]. In fact, the risk of hypoglycemia is a barrier to optimal treatment of type 1 diabetes (T1DM) and type 2 diabetes (T2DM), especially within the context of insulin therapy [5].

Hypoglycemic episodes may manifest in different ranges from asymptomatic to severe neurological symptoms, like dizziness, confusion, weakness and loss of consciousness [6–8]. Recurrent asymptomatic episodes characterizes hypoglycemia unawareness, which has been shown to increase the risk of severe hypoglycemic episodes [6, 9]. Moreover, patient fear of hypoglycemia is frequent and may also affect treatment adherence and, thus, glycemic control [6, 10].

Some observational studies have reported that individuals with T1DM experience about 42.0–136.8 non-severe hypoglycemic events per patient-year [11, 12]. Regarding T2DM, non-severe hypoglycemia seems to occur less frequently (0.2 to 48.0 events per patient-year) but, like in T1DM, may increase with longer duration of insulin therapy [11, 12]. Nonetheless, most studies had a retrospective/cross-sectional design, and are almost limited to North American and European health contexts [12].

The Hypoglycemia Assessment Tool (HAT) study was a multinational non-interventional study that included 27,585 adult T1DM or T2DM patients, from 24 countries in six world regions. The study results have shown that, during a 4-week prospective period, 83.0% T1DM patients and 46.5% T2DM patients had at least one hypoglycemic episode [12]. However, the study initially did not include Brazil, one of the top 10 countries in number of people living with diabetes: it was estimated that 11.9 million Brazilian adults were living with diabetes in 2013, which correspond to a prevalence of 11.9% [13].

Recognizing the scarce data about hypoglycaemia and how this affects diabetes control and management, the HAT study was implemented in Brazil to determine the proportion of patients experiencing hypoglycemic events, to characterize patient awareness, fear and attitudes towards hypoglycemia, and to estimate health resource use and costs of managing hypoglycemic events among patients with T1DM or insulin-treated T2DM.

## Methods

### Patients and study design

This was a national, non-interventional, multicenter study, with a 6-month retrospective and a 4-week prospective evaluation of hypoglycemic events. The primary endpoint was defined as the percentage of patients experiencing at least one hypoglycemic event during the 4-week prospective period. Included patients were ≥ 18 years-old and had T1DM or T2DM treated with insulin for more than 12 months. Patients were excluded from the study if followed-up at non-ambulatory setting, or not able to answer to the written survey, or if participating in any interventional clinical study. All participants provided their written informed consent prior to any study-related activity. Eligible participants were consecutively enrolled from 21 centers in Brazil, when attending to a routine medical appointment with a diabetes specialist, between October 2014 and February 2015.

### Data collection

At baseline, the first questionnaire recorded baseline demographic and treatment information, and evaluate patient knowledge, hypoglycemia unawareness and perceptions of hypoglycemia over the last 6 months. History of severe hypoglycemic events during the same period was also collected, as well as history of symptomatic events over the last 4 weeks. A patient diary was provided to each participant, to register hypoglycemic events during the prospective period. A second questionnaire evaluated the history of both severe and symptomatic hypoglycemia over the 4 weeks after baseline. When a patient recorded more hypoglycemic events in the diary than during the second questionnaire, the patient diary information was used to estimate prevalence and hypoglycemia rates.

### Hypoglycemia definitions

Severe hypoglycemia was defined according to the American Diabetes Association definition, as any hypoglycemic event requiring assistance of another person to actively administer carbohydrate, glucagon, or other resuscitative actions [14]. Non-severe hypoglycemia was defined as events managed by the patient alone, and “any hypoglycemia” resulted from the sum of severe and non-severe hypoglycemia. Other definitions used in this study were nocturnal hypoglycemia (hypoglycemia occurring between midnight and 06:00 h) and hypoglycemia requiring hospital admission.

### Hypoglycemia unawareness and fear of hypoglycemia

Hypoglycemia unawareness was evaluated with the question ‘Do you have symptoms when you have a low sugar level?’, where ‘always’ and ‘usually’ denoted normal, ‘occasionally’ denotes impaired, and ‘never’ corresponded to severely impaired awareness. Fear of hypoglycemia was reported on a 10-point scale, from ‘not afraid at all’ to ‘absolutely terrified’.

### Use of health resources and costs

Self-reported use of healthcare resources, related with hypoglycemic events, was collected from the patient diary, with data from the 4-week prospective period. The economic analysis assumed the societal perspective, with the estimates of direct cost based on the healthcare resources required to manage hypoglycemic events (i.e. hospitalization, emergency assistance, extra visits to physician/nurse and outpatient administration of carbohydrates or glucagon), and indirect costs relate with labor absenteeism due to hypoglycemia (i.e., sick leave and delays to work) calculated considering the Capital Human Approach using Brazilian minimum monthly income by gender and age class. Prices for health resources were obtained from official Brazilian sources DATASUS—*Base de dados ambulatorial e hospitalar* 2014 (hospitalization and emergency visits), SIGTAP—*Sistema de Gerenciamento da tabela de Procedimentos*, *Medicamentos e OPM do SUS* (medical visits) and BPS—*Banco de Preços em Saúde* (use of glucagon). Yearly costs were extrapolated based on the prospective assessment, in Brazilian Reais, for the year 2015.

### Statistical analysis

Sample size was calculated based on the primary endpoint of the study. The inclusion of at least 675 patients would enable the estimation of 95% confidence interval (CI) for the percentage of patients experiencing at least one hypoglycemic event during the observation period, with a maximum precision error of 4% and assuming a drop-out rate of 11%.

The incidence of hypoglycemic episodes during prospective period was defined as the percentage of patients experiencing at least one hypoglycemic event during the 4-week observational prospective period. Patients with hypoglycemic events were identified if having reported at least one hypoglycemic event in either the second questionnaire or the patient diary. The incidence of hypoglycemic events, by diabetes type (T1DM or T2DM), was calculated with corresponding 95% CI assuming a binomial distribution. For secondary endpoints, the incidence of various types of hypoglycemia was calculated as the number of events per patient year, as expressed by [total number of events/total follow-up time (patient years)]. The frequency of patients with at least one severe, non-severe, any, nocturnal and hospital-requiring hypoglycemic events, are also presented by diabetes type, and stratified by glycated hemoglobin (HbA1c) at baseline and by age (< 65 years old versus ≥ 65 years old). In addition, the Mann–Whitney test was performed to examine the association between HbA1c and the occurrence of severe hypoglycemia.

To examine the relationship between hypoglycemia and eventual predictors, negative binomial regression models were defined by specifying a log-transformed exposure time offset term and adjusting (stepwise) for all variables in the model, namely: age (years), gender, HbA1c (%), type of diabetes, duration of diabetes (years), duration of insulin therapy (years), type of insulin therapy, frequency of blood glucose testing, hypoglycemia unawareness, knowledge of hypoglycemia before baseline, fear of hypoglycemia, study period (prospective/retrospective) and diabetes type. No imputation of missing data was performed. The negative binomial regression model for the hospital-requiring hypoglycemic events was not presented, as some convergence criteria were not satisfied.

Of those patients who had ever experienced hypoglycemia, the number and proportion with normal, impaired, and severely impaired hypoglycemia awareness, were summarized by diabetes type and by whether or not patients have experienced severe hypoglycemia in the 6 months before baseline. A logistic regression model was performed to examine the relationship between the above mentioned predictor variables and the odds of having an impaired or severely impaired hypoglycemia unawareness.

Patients’ fear of hypoglycemia was also analyzed by diabetes type and whether or not patients have experienced severe hypoglycemia in the 6 months before baseline. The number and proportion of patients who, as a result of fear of hypoglycemia, have and have not consulted their doctor/nurse, increased calorie intake, avoided physical exercise, reduced insulin doses, skipped insulin injections and increased blood glucose monitoring was also determined.

The study was conducted in accordance with the principles of the Declaration of Helsinki and of the Guidelines for Good Pharmacoepidemiology Practices. The study protocol and patient informed consent documents were reviewed and approved by the ethics committees of all study centers.

## Results

### Sample characteristics

From 679 included patients, 321 had T1DM, 293 T2DM and 65 had unclassified T1DM/T2DM. Table 1 presents the main characteristics of the study sample. Patients with T1DM were younger than those with T2DM (median age 33.0 years vs. 62.0 years) and more T1DM patients were full-time employees (45.5% vs. 21.2%). Both T1DM and T2DM patients had a similar duration of diabetes (both with a median of 15.0 years) but T1DM patients had a longer duration of insulin use (median of 14.0 years vs. 6.0 years). The most common treatment (77.9%) for T1DM was both short and long-acting insulin analogues, used alone or in combination with other treatments, while oral anti-diabetes drugs combined with insulin was the most frequent option (60.4%) for T2DM. Among T1DM patients that had a previous experience of hypoglycemia (n = 313), 42.6% identified hypoglycemic events by both symptoms and blood glucose measurement. Regarding T2DM patients with a previous experience of hypoglycemia (n = 247), 41.4% reported using only symptoms to identify hypoglycemia. A total of 588 (86.6%) patients completed the second questionnaire and the patient diary, from which 276 were T1DM patients, 254 were T2DM patients and 58 had unclassified T1DM/T2DM.

**Table 1:** Demographics and diabetes history of study population

	T1DM (n = 321)	T2DM (n = 293)
Female sex, n (%)	188 (58.8%)	162 (55.5%)
Age (years), median [Q1, Q3]	33.0 [26.0, 44.0]	62.0 [55.0, 68.5]
Full-time employment, n (%)	136 (45.5%)	58 (21.2%)
Duration of diabetes (years), median [Q1, Q3]	15.0 [9.0, 22.0]	15.0 [10.0, 22.0]
Last measured HbA1c value (%), median [Q1, Q3]	7.7 [7.0, 8.5]	7.9 [7.3, 9.1]
Checks blood sugar levels, n (%)	314 (98.4%)	259 (88.7%)
Has ever experienced hypoglycemia, n (%)	313 (97.5%)	247 (84.3%)
(if yes) Patients identifying hypoglycemia based on, n (%)		
Symptoms only	83 (27.4%)	96 (41.4%)
Blood glucose measurement only	23 (7.6%)	38 (16.4%)
Either symptoms or a blood glucose measurement	56 (18.5%)	28 (12.1%)
Both symptoms and blood glucose measurement	129 (42.6%)	65 (28.0%)
Other	12 (4.0%)	5 (2.2%)
Missing	18	61
Duration of insulin use (years), median [Q1, Q3]	14.0 [8.0, 22.0]	6.0 [3.0, 10.0]
Diabetes treatment, n (%)^a^		
Only short-acting insulin	26 (8.2%)	15 (5.1%)
Only long-acting insulin	14 (4.4%)	123 (42.0%)
Both short- and long-acting insulin	250 (77.9%)	137 (46.8%)
Mixed insulin	12 (3.8%)	11 (3.8%)
Insulin pump	17 (5.3%)	1 (0.3%)
Oral anti-diabetes treatment	29 (9.1%)	177 (60.4%)
Injectable anti-diabetes treatments other than insulin	44 (13.8%)	4 (1.4%)
Patients who completed second questionnaire	276 (86.0%)	254 (86.7%)

*Q1* lower quartile, *Q3* upper quartile

^a^Each patient can use more than one treatment for diabetes

### Incidence of hypoglycemia

In the 4-week prospective period, 253 (91.7%; 95% CI 88.4–94.9%) T1DM patients and 157 (61.8%; 95% CI 55.8–67.8%) T2DM patients reported at least one hypoglycemic event, based on the second questionnaire or patient diary. For the same period, Table 2 presents the frequency and incidence rates of severe, non-severe, any, nocturnal and requiring hospitalization hypoglycemic events, considering only the second questionnaire information. Regarding severe hypoglycemia, 25.7% T1DM patients and 13.4% T2DM patients had at least one episode, with estimated incidence rates of 9.8 and 6.2 events/patient-years. A total of 5.2% T1DM (vs. 3.3% T2DM) patients reported at least one episode requiring hospitalization, with annual rates of 1.6 (vs. 0.4) events/patient-years. T1DM patients also reported higher frequency of nocturnal event (54.9% vs. 27.4%) and higher incidence rates for these hypoglycemic episodes (23.6 vs. 6.1 events/patient-years). Asymptomatic hypoglycemic events were reported in 20.6% (n = 66) T1DM and 10.6% (n = 31) T2DM patients.

**Table 2:** Frequency and incidence of severe, non-severe, any, nocturnal and hospital-requiring hypoglycemic events, 4 weeks after baseline

	T1DM (n = 276)	T2DM (n = 254)
Severe		
Frequency, n (%)	71 (25.7%)	34 (13.4%)
Incidence rate, events/patient-years (95% CI)	9.8 (8.5–11.3)	6.2 (5.2–7.4)
Non-severe		
Frequency, n (%)	235 (85.1%)	128 (50.4%)
Incidence rate, events/patient-years (95% CI)	99.0 (94.8–103.3)	25.5 (23.3–27.9)
Any		
Frequency, n (%)	248 (89.9%)	142 (55.9%)
Incidence rate, events/patient-years (95% CI)	108.8 (104.4–113.3)	31.7 (29.3–34.3)
Nocturnal		
Frequency, n (%)	141 (54.0%)	62 (27.4%)
Incidence rate, events/patient-years (95% CI)	23.6 (21.4–25.9)	6.1 (4.9–7.4)
Requiring hospitalization		
Frequency, n (%)	14 (5.2%)	8 (3.3%)
Incidence rate, events/patient-years (95% CI)	1.6 (1.1–2.3)	0.4 (0.2–0.8)

*CI* confidence interval

### Factors associated with hypoglycemia

The risk of severe, non-severe and any hypoglycemia were significantly higher during the prospective period (Table 3).

**Table 3:** Negative binomial regressions for severe, non-severe, any, and nocturnal hypoglycemic events

	IRR (95% CI)	p-value
Severe hypoglycemic events^a^		
Age (years)	0.98 (0.97–0.99)	0.001
Gender: female [ref. male]	1.67 (1.01–2.77)	0.047
Duration of diabetes (years)	1.03 (1.01–1.05)	0.001
Fear of hypoglycemia (scale 1–10)	1.17 (1.06–1.29)	0.002
Period: prospective [ref. retrospective]	4.75 (2.75–8.23)	< 0.001
Non-severe hypoglycemic events^a^		
Duration of insulin therapy (years)	1.02 (1.01–1.03)	0.003
Diabetes type: T1DM [ref. T2DM]	2.59 (1.99–3.38)	< 0.001
Frequency of blood glucose monitoring (days)	1.14 (1.07–1.20)	< 0.001
Period: prospective [ref. retrospective]	1.81 (1.53–2.14)	< 0.001
Any hypoglycemic events^a^		
Duration of insulin therapy (years)	1.02 (1.01–1.03)	< 0.001
Diabetes type: T1DM [ref. T2DM]	2.34 (1.81–3.03)	< 0.001
Frequency of blood glucose monitoring (days)	1.13 (1.06–1.20)	< 0.001
Period: prospective [ref. retrospective]	2.05 (1.76–2.39)	< 0.001
Nocturnal hypoglycemic events^a^		
Diabetes type: T1DM [ref. T2DM]	2.06 (1.46–2.90)	< 0.001
Frequency of blood glucose monitoring (days)	1.12 (1.05–1.20)	0.001
Hypoglycemia awareness: impaired or severely impaired [ref. normal]	0.65 (0.46–0.91)	0.012

* *Negative binomial regression model (after stepwise)* Subject effect: Patient; Within-subject effect: Period; Offset variable: Ln (follow-up time); Dependent variable: number of hypoglycemic events. Independent variables: age, gender, HbA1c, diabetes type, duration of diabetes, duration of insulin therapy, type of insulin therapy, frequency of blood glucose monitoring, knowledge of hypoglycemia, hypoglycemia unawareness, fear of hypoglycemia and period

*IRR* incidence rate ratio, *CI* confidence interval. *Ref.* reference category versus the one is making comparison (categorical variables); for continuous variables, please consider IRR per additional unit

The risk of severe hypoglycemic events was higher for female patients (IRR = 1.67), patients with longer duration of diabetes (IRR = 1.03) and with more fear of hypoglycemia (IRR = 1.17), while decreasing with age (IRR = 0.98). Regarding non-severe and any hypoglycemia, patients with higher frequency of blood glucose monitoring, longer duration of insulin therapy, and type 2 diabetes had higher risk. The risk of nocturnal hypoglycemic events was also significantly higher for patients with higher frequency of blood glucose monitoring (IRR = 1.12), for T2DM patients (IRR = 2.06), and associated with impaired hypoglycemia awareness (IRR = 0.65).

**Fig. 1:**
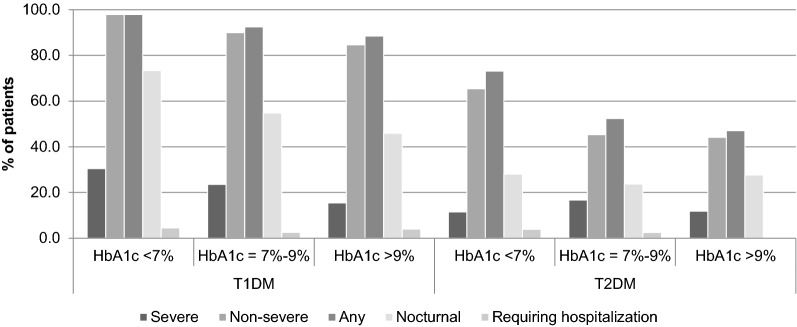
Patients with severe, non-severe, any, nocturnal and hospital-requiring hypoglycemic events, stratified by glycated hemoglobin (HbA1c) at baseline and by diabetes type

**Fig. 2:**
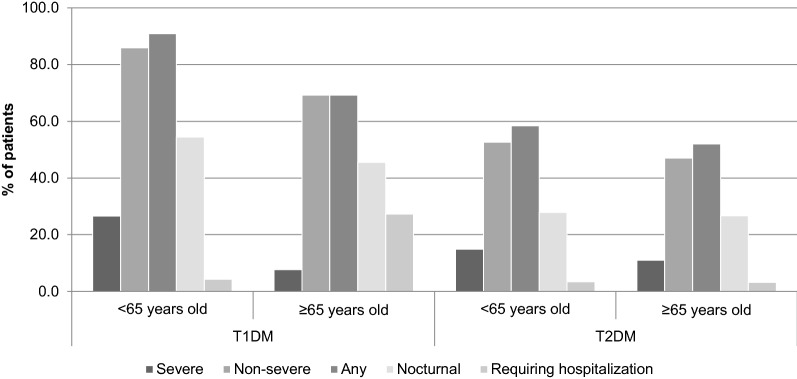
Patients with severe, non-severe, any, nocturnal and hospital-requiring hypoglycemic events, stratified by age (< 65 years old versus ≥ 65 years old) at baseline and by diabetes type

Glycated hemoglobin (HbA1c) at baseline was not associated with risk of hypoglycemia after adjusting for potential confounders, although a negative association was found between HbA1c and severe hypoglycemia (Mann–Whitney: p-value = 0.003). In addition, T1DM and T2DM patients with HbA1c < 7% at baseline reported more severe, non-severe, any, nocturnal and hospital-requiring hypoglycemic events than those between 7 and 9% or higher than 9% (Fig. 1). Similarly, for both diabetes types, patients younger than 65 years old reported more severe, non-severe, any and nocturnal events when compared with older patients (Fig. 2). On the contrary, for T1DM patients the occurrence of hypoglycemia requiring hospitalization was higher in older patients when compared with patients who were less than 65 years of age.

### Hypoglycemia awareness

**Fig. 3:**
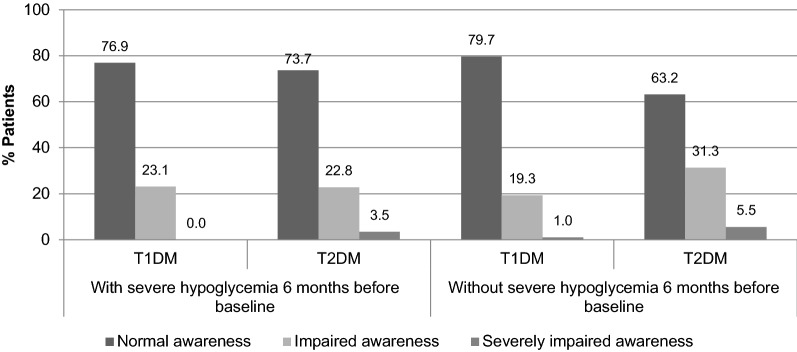
Hypoglycemia unawareness at baseline, by diabetes type and by previous experience of severe hypoglycemia in the last 6 months

At baseline, 21.1% T1DM and 34.6% T2DM patients had impaired or severely impaired awareness. When comparing patients that had experienced or not hypoglycemic events in the 6 months before baseline (Fig. 3), T2DM patients with previous hypoglycemia seem to be more aware of hypoglycemia than those without previous experience (73.7% vs. 63.2%), while similar results were observed among T1DM patients with and without previous hypoglycemia (76.9% vs. 79.7%). In addition, the chance of having an impaired or severely impaired hypoglycemia unawareness decreased with higher age (OR 0.98, 95% CI 0.97–0.99), after adjusting for gender, HbA1c, duration/type of diabetes and insulin therapy, frequency of blood glucose monitoring, knowledge and fear of hypoglycemia, psychosocial well-being, severe and non-severe hypoglycemia events.

### Fear of hypoglycemia

The mean score of hypoglycemia fear was 5.9 ± 3.1 in T1DM and 5.4 ± 3.9 in T2DM. The most common actions after a hypoglycemic event were to increase calorie intake (60.3%) and blood glucose monitoring (58.0%), and to reduce or skip insulin doses (30.8%). Table 4 presents the most common actions after a hypoglycemic event, by diabetes type and by previous experience of severe hypoglycemia in the last 6 months. A higher proportion of T1DM patients that experienced severe hypoglycaemia (compared to T2DM patients) have consulted their physician/nurse (60.2% vs. 48.3%), increased calorie intake (74.4% vs. 63.3%), avoid physical activity (37.3% vs. 27.3%), reduced insulin doses (91.6% vs. 44.1%) and increased the number of times checking blood glucose (76.5% vs. 50.8%). Regarding skipping insulin injections, similar results were observed between T1DM and T2DM patients (33.3% and 31.0%, respectively). When considering patients without severe hypoglycaemia in the last 6 months, more T1DM (vs. T2DM) patients consulted their physician (43.5% vs. 28.4%), increased calorie intake (67.5% vs. 51.4%), avoid physical exercise (23.3% vs. 13.5%), reduced insulin doses (64.5% vs. 20.2%), skipped insulin doses (24.3% vs. 18.9%), or increased the number of times checking blood glucose (70.5% vs. 49.1%).

**Table 4:** Fear of hypoglycemia and actions taken after hypoglycemic events, by diabetes type and previous experience of severe hypoglycemia

	T1DM		T2DM	
	With severe hypoglycemia in the last 6 months (n = 119)	Without severe hypoglycemia in the last 6 months (n = 198)	With severe hypoglycemia in the last 6 months (n = 60)	Without severe hypoglycemia in the last 6 months (n = 231)
Fear of hypoglycemia at baseline, mean ± SD^a^	6.6 ± 2.97	5.4 ± 3.00	6.5 ± 3.40	5.1 ± 3.99
Actions taken after hypoglycemic events, n (%)^b^				
Consulted their physician/nurse	68 (60.2%)	80 (43.5%)	28 (48.3%)	61 (28.4%)
Increased calorie intake	87 (74.4%)	129 (67.5%)	38 (63.3%)	113 (51.4%)
Avoid physical exercise	41 (37.3%)	42 (23.3%)	15 (27.3%)	28 (13.5%)
Reduced insulin doses	93 (91.6%)	120 (64.5%)	26 (44.1%)	43 (20.2%)
Skipping insulin injections	37 (33.3%)	44 (24.3%)	18 (31.0%)	40 (18.9%)
Increased the number of times checking blood sugar	88 (76.5%)	134 (70.5%)	30 (50.8%)	108 (49.1%)

*SD* standard-deviation

^a^On a scale from 0 to 10, where 0 = Not afraid at all and 10 = absolutely terrified

^b^More than one possible answer

### Costs of managing hypoglycemic events

A total of 281 patients (T1DM/T2DM: 47%/53%) had no missing data in health resource variables and were considered for an economic analysis (Table 5). On average, hypoglycemic episodes represented a yearly cost to the Brazilian society of R$709 per T1DM patient (range R$0–R$12.364; direct cost: R$640; indirect cost: R$69) and R$396 per T2DM patient (range R$0–R$10.431; direct cost: R$390; indirect cost: R$6). Hospitalizations were the main cost driver.

**Table 5:** Health resource use and productivity loss due to hypoglycemia (n = 281)

	T1DM (n = 131)	T2DM (n = 150)
Monthly health resource use		
Hospitalization, %	5.3	2.7
Mean duration (days)	4	4
Emergency visits, %	3.1	4.7
Mean number of emergency visits	3	2
Physician/nurse visits, %	0.8	2.7
Mean number of visits	3	3
Use of glucagon, %	19.8	12
Work productivity loss		
Patients with lost working days, %	3.2	0.7
Mean number of lost working days	3.0	2.4
Yearly cost per patient (R$)	709	396
Yearly cost per component, %		
Hospitalization	63.8	65.2
Emergency visits	3.8	7.3
Physician/nurse visits	0.6	0.8
Use of glucagon	22.0	25.3
Productivity loss	9.7	1.5

*SD* standard-deviation

## Discussion

The HAT study in Brazil evaluated the frequency and incidence rate of hypoglycemia in a cohort of patients with insulin-treated diabetes mellitus. It was found that 91.7% T1DM patients and 61.8% T2DM patients had at least one hypoglycemic event during the 4 weeks after baseline, a short period of observation. These frequencies were higher than those observed at the global HAT study, where, overall, 83.0% T1DM and 46.5% T2DM patients had at least one hypoglycemia in the prospective period [12]. In addition, hypoglycemic rates observed in Brazil were even higher that those reported by the global HAT study for the Latin America region (87.4% T1DM and 43.8% T2DM).

The incidence of severe hypoglycemia was higher in the present study, with 25.7% T1DM patients and 13.4% reporting at least one event during the prospective period. In the global study, the proportions were 14.4% and 8.9%, respectively [12]. The same trend was observed for the incidence rates: 4.9 and 2.5 severe events per patient-year in the global study vs. 9.8 and 6.2 severe events per patient-year in this study, for T1DM and T2DM patients, respectively. When considering nocturnal episodes, Brazilian patients also reported higher incidence rates (23.6 and 6.1 events/patient-years among T1DM and T2DM, respectively) compared to the rates observed in the global study (11.3 and 3.7 events/patient-years among T1DM and T2DM, respectively).

The higher hypoglycemia frequencies that were observed, especially among T2DM patients, may reflect differences in diabetes management, even between countries from the same geographic region. A cross-sectional study that included T1DM Brazilian patients reported that 96.8% experienced hypoglycemia at least once in the last 3 months and that 89.6% had at least one hypoglycemic event in the last month [15].

Predictors of having any hypoglycemia included the higher frequency of blood glucose monitoring, longer duration of insulin therapy, and T1DM. Longer duration of insulin therapy has been described as a predictor of hypoglycemic events, and may reflect the need for treatment adjustment that occurs with the natural course of the disease and counter regulatory hormonal deficiencies related to pancreatic beta-cell failure [8, 16]. The higher frequency of blood glucose monitoring was also associated with non-severe hypoglycemic events, probably because some of these events were asymptomatic and only identified by blood glucose measurement.

At baseline, about 20% T1DM patients and 35% T2DM patients had impaired or severely impaired hypoglycemia awareness. We hypothesize that T1DM patients could be more knowledgeable about hypoglycemia and monitor glycemia more frequently, even when asymptomatic. Hypoglycemia unawareness was associated with lower rates of reported nocturnal hypoglycemia, which should be further evaluated since prolonged nocturnal events can lead to disturbances of cardiac function [17]. Unlike other studies that report an association between the hypoglycemia unawareness with older T2DM patients [9, 18], the results herein obtained suggested that older patients might have a slightly decreased hypoglycemic unawareness than younger patients. Nevertheless, further studies are necessary in order to clarify this association, while adjusting to other confounding variables that may have not been considered in the present study.

Similar to other studies, fear of hypoglycemia was associated with higher risk of severe hypoglycemia, most probably due to reverse causality of previous severe events [12]. Overall, patients from our study seem to experience more fear than that observed in the global study, with mean scores of 5.9 vs. 4.7 for T1DM patients, and 5.4 vs. 4.4 for T2DM patients [19]. In the prospective period, the more frequent reaction following hypoglycemic events was to increase blood glucose monitoring (63.8% T1DM patients and 50.9% T2DM patients). In the global study, this reaction was also the most frequent among T1DM patients (69.7%), but requiring any form of medical assistance was the most frequent reaction among T2DM patients (62.0%) [19]. Furthermore, our study showed that 91.6% T1DM patients with a previous severe hypoglycemia reported the reduction of insulin doses, a reaction that may compromise glycemic control.

Estimated costs for hypoglycaemias in T1DM patients are almost two times the costs in T2DM, most probably due to higher incidence of hypoglycaemias and need of hospitalizations [20]. Of notice, results of the HAT study for Central and Eastern European countries have reported that T1DM patients had less hospitalizations due to hypoglycaemia compared to T2DM patients (1.2% vs. 2.1%) [21]. Another study from Canada also confirm hospital admissions as the main cost driver but reported a higher cost of managing hypoglycemias among T2DM patients, as a result of more medical appointments and more lost working days in this group [22]. These observations, alongside with the high incidence rates of hypoglycemia, reinforce the need of optimizing patient access to healthcare, in order to achieve a better tailored insulin regimen and glycemic control and avoid the need of hospitalizations.

As a non-interventional self-reported study, there were some limitations such as potential misunderstanding of questions, and erroneous self-perception of hypoglycemic events by the patient, besides the possible recall bias, especially for baseline questions related to the previous 6 months. Missing data was frequent in some questions, namely when patients who reported having hypoglycemic events in the patient diary did not report the number of events that occurred in second questionnaire. When discrepancies were found between patient diary and second questionnaire regarding the registries of hypoglycemia, it was assumed the highest frequency stated on either of these forms to estimate frequency and incidence rate but the higher reporting in the patient diary may be partially explained by an increased awareness about hypoglycemia and the reminder effect of the patient diary.

The HAT study methodology enables the comparison of data across several regions and countries, thus providing real-world information about hypoglycemia rate and risk factors. The self-reporting of hypoglycemia, although prone to misunderstanding and overestimation of hypoglycemia, provides the collection of events that patients could, otherwise, forgot to report or neglect due to the absence of symptoms. The evaluation of several patient-centered dimensions, such as hypoglycemia awareness and fear, is expected to support clinicians on tailoring insulin treatment and improving patient education and diabetes management.

## Conclusions

In Brazil, the HAT study highlighted that hypoglycemic events are frequent among patients with insulin-treated diabetes and may compromise patient adherence to insulin treatment [23]. Alongside the Brazilian high prevalence of diabetes and short and long-term consequences of hypoglycemia, these findings require a further evaluation of diabetes management strategies and barriers to a more effective and safe control.

## Abbreviations

CI: confidence interval
HAT: Hypoglycemia Assessment Tool study
HbA1c: glycated hemoglobin
IRR: incidence rate ratio
Q1: lower quartile
Q3: upper quartile
ROC: receiver operating characteristic curve
SD: standard-deviation
T1DM: type 1 diabetes mellitus
T2DM: type 2 diabetes mellitus
